# Mutations in Tau Protein Promote Aggregation by Favoring Extended Conformations

**DOI:** 10.1021/jacsau.3c00550

**Published:** 2023-12-19

**Authors:** Kevin Pounot, Clara Piersson, Andrew K. Goring, Frédéric Rosu, Valérie Gabelica, Martin Weik, Songi Han, Yann Fichou

**Affiliations:** † Univ. Grenoble Alpes, CEA, CNRS, Institut de Biologie Structurale, 38000 Grenoble, France; ‡ Univ. Bordeaux, CNRS, Bordeaux INP, CBMN, UMR 5248, IECB, 33600 Pessac, France; § Department of Chemistry and Biochemistry, University of California Los Angeles, Los Angeles, California 90095, United States; ∥ Univ. Bordeaux, CNRS, INSERM, IECB, UAR3033, US01, F-33600 Pessac, France; ⊥ Univ. Bordeaux, CNRS, INSERM, ARNA, UMR5320, U1212, IECB, 33600 Pessac, France; # Department of Chemical Engineering, University of California Santa Barbara, Santa Barbara, California 93106, United States; ∇ Department of Chemistry and Biochemistry, University of California Santa Barbara, Santa Barbara, California 93106, United States

**Keywords:** intrinsically disordered protein, SAXS, tau protein, amyloid, IDP conformation

## Abstract

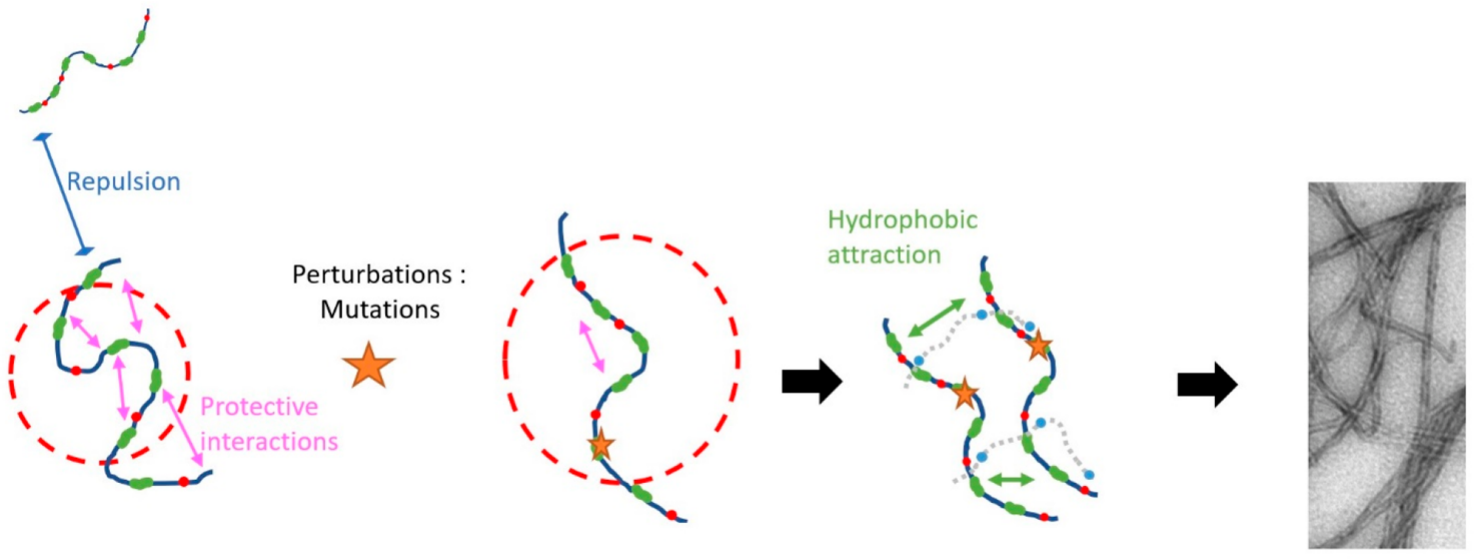

Amyloid aggregation of the intrinsically disordered protein (IDP) tau is involved in several diseases, called tauopathies. Some tauopathies can be inherited due to mutations in the gene encoding tau, which might favor the formation of tau amyloid fibrils. This work aims at deciphering the mechanisms through which the disease-associated single-point mutations promote amyloid formation. We combined biochemical and biophysical characterization, notably, small-angle X-ray scattering (SAXS), to study six different FTDP-17 derived mutations. We found that the mutations promote aggregation to different degrees and can modulate tau conformational ensembles, intermolecular interactions, and liquid–liquid phase separation propensity. In particular, we found a good correlation between the aggregation lag time of the mutants and their radii of gyration. We show that mutations disfavor intramolecular protein interactions, which in turn favor extended conformations and promote amyloid aggregation. This work proposes a new connection between the structural features of tau monomers and their propensity to aggregate, providing a novel assay to evaluate the aggregation propensity of IDPs.

## 1 Introduction

Tau pathologies form a class of neurodegenerative diseases in which deleterious deposits enriched in a protein called tau are present in the brain. The tau protein accumulates in these deposits in the form of amyloid filaments, which are highly ordered protein aggregates in which each protein stacks in a cross-beta structure. Strikingly, recent structural work pointed toward a correlation between the conformation of tau within these amyloid aggregates and the associated pathology phenotype.^1^

Several disease-associated mutations have been identified in the tau proteins, in particular in frontotemporal dementia and parkinsonism (FTDP) linked to chromosome 17 (FTDP-17) (see review by Goedert and Jakes).^2^ Most of the mutations are present in the repeat domains, i.e., in the region that both binds to microtubule and forms the core of amyloid filaments. Accordingly, mutations can exhibit a dual effect on the protein activity: they hinder microtubule binding,^3−5^ and they promote amyloid assembly.^5−8^ The latter findings result from studies conducted under different conditions (inducers, incubation, and buffer) and are thus not amenable to a quantitative comparison across mutants. The lack of consistency makes the evaluation of the effect of a specific mutation difficult. Tau is an intrinsically disordered protein (IDP), meaning that it does not possess a well-defined 3D structure but rather coexists with many different conformations. The conformational landscape of IDPs is highly complex leading to multiple aggregation pathways.^9^ Therefore, linking structural properties with aggregation is not trivial. Using different aggregation enhancers (pH, cofactors), experimental studies showed that aggregation prone states of IDPs can be characterized by global collapse for protein prothymosin-α^10^ or by local compaction and extension for α-synuclein and tau, respectively.^11,12^ In solution state, tau lacks any stable secondary structure elements and is mostly in a random coil conformation.^13^ This limits the applicable biophysical methods and has hindered the understanding of the relationship between tau conformations and the tau aggregation propensity. The mutation P301L was first shown by NMR to have a small but significant effect on the local conformation with no increased beta-sheet propensity.^14^ Early work suggested that mutations modify the conformation of the flanking regions of aggregation prone regions PHF6 (306-311) and PHF6*(275–280).^6^ More details were provided by Chen et al. showing that unshielding the PHF6 region can explain mutation-induced aggregation enhancement.^15^ More generally, long-range intramolecular interactions seem to play an important role in aggregation modulation as shown by FRET and cross-linking mass spectrometry.^15−17^ Yet, there is no quantitative work linking the modulation of aggregation propensity by mutations and their structural properties.

**Figure 1:**
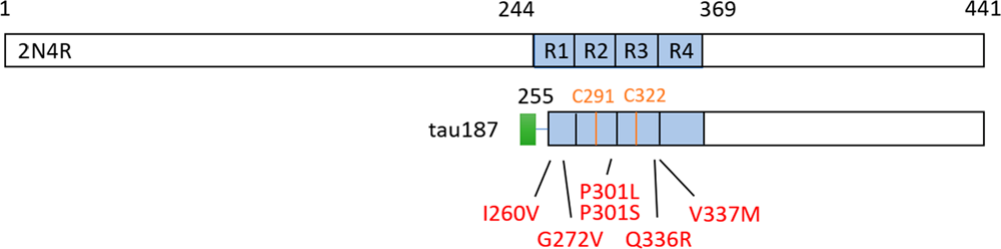
Longest human tau isoform, 2N4R contains 441 amino acids. In this work we have used a fragment of 2N4R that starts at residue 255, referred to as tau187, onto which were added single-point mutations. Each mutation (red) is located in one of the repeat domains R1–R4. The green region indicates a poly histidine tag.

Here we studied the effect of six different disease-associated mutations on the aggregation propensity and structural features of tau. We used a fragment of tau, referred to as tau187 (residues 255–441 of full length 2N4R, Figure 1), onto which we engineered the following single-point mutations: I260V, G272V, P301L, P301S, Q336R, and V337M (Figure 1). Tau187 contains most of the four repeat domains and the C-terminal region, thus encompassing the known amyloid cores, while being more prone to aggregate as compared to full-length2N4R. Using small-angle X-ray scattering (SAXS), native mass spectrometry (MS), and biochemical characterization methods, we show that a single-point mutation modulates tau monomer conformation, intra- and interprotein interactions and aggregation propensity. We find a good correlation between aggregation lag time and the radius of gyration.

## 2 Results

From the construct tau187, termed tau187 WT, we made six mutants that each contained a disease-associated single-point mutation: tau187-I260V, tau187-G272V, tau187-P301L, tau187-P301S, tau187-Q336R, and tau187-V337M (Figure 1).

### 2.1 Single-Point Mutations Have an Important Effect on Aggregation Kinetics

Amyloid aggregation for all mutants was assessed by thioflavin T (ThT) fluorescence and transmission electron microscopy (TEM). All mutants were stable over 4 days at 37 °C under shaking (Figure S1). The addition of a cofactor, the RNA homonucleotide polycytosine (polyC), was used to favor the formation of amyloid fibrils on the experimental time scale. Aggregates were observed by TEM for all mutants (Figure S2).

**Figure 2:**
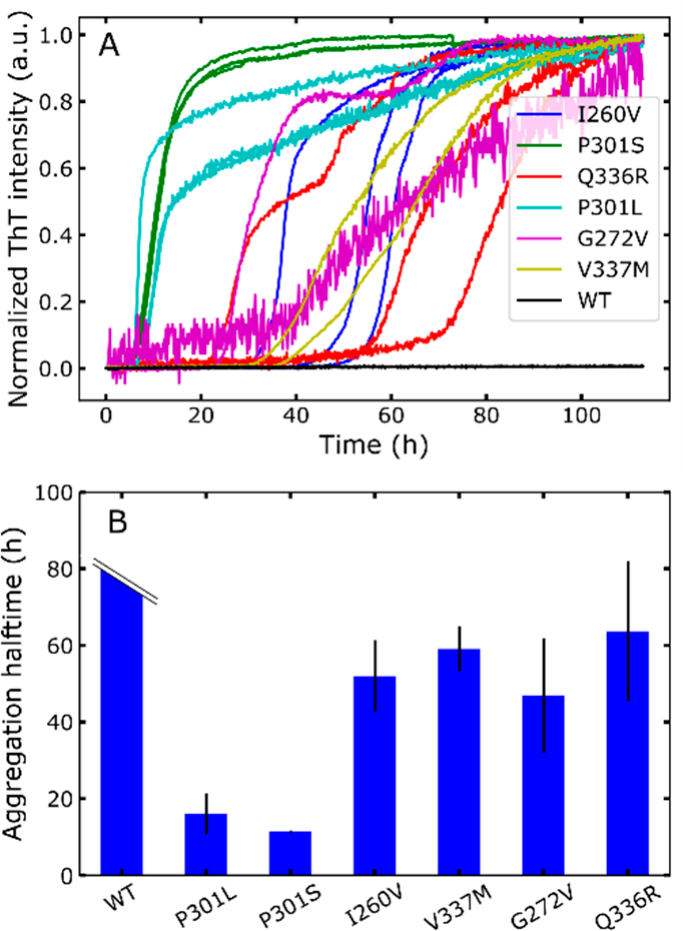
ThT fluorescence as a function of time for different mutants of tau187 incubated with RNA polyC (A). The curves are normalized between 0 and 1. Aggregation halftime for each mutant (B) extracted from a fit of each ThT curve. Error bars represent the standard deviation over the halftimes obtained from the different replicates. Protein and RNA concentrations were 20 and 200 μM, respectively.

We assessed the aggregation propensity by recording the ThT fluorescence as a function of time, where the RNA was added at time *t* = 0 h (Figure 2A). All ThT curves were fitted with a sigmoid function (eq 2) from which we extracted the aggregation halftime (Figure 2B). The fitting functions are plotted in Figure S3. The single-point mutations have a drastic effect on aggregation kinetics. All mutations enabled aggregation to occur over 110 h, which was not seen in the WT version. The P301S and P301L mutations lead to the fastest aggregation. The same experiments were performed with other nucleotides (polyA and polyU) and lead to the same qualitative trend (Figure S4A–D)

## 2.2 Tau Expansion, Due to Mutations, Correlates with Aggregation Propensity

**Figure 3:**
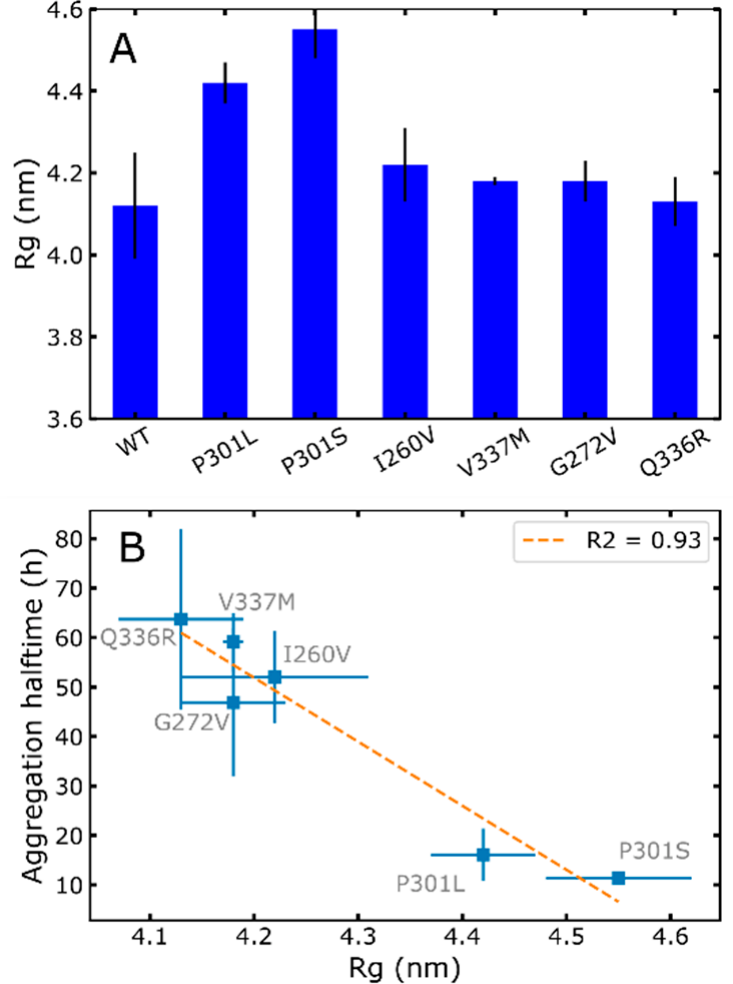
A single-point mutation significantly changes tau conformational ensembles, as shown by different radii of gyration *R*_g_ (A). *R*_g_ correlates with aggregation halftime (B). Error bars on *R*_g_ originates from the covariance matrix generated by the fitting procedure. Tau187-WT does not aggregate on the observed time scale and thus has no aggregation halftime. Hence it is not plotted here in panel B.

We then characterized the structural properties of each mutant by small-angle X-ray scattering (SAXS). We measured the SAXS of all tau187 mutants and extracted their radius of gyration (Figure 3A). tau187-WT exhibits the smallest *R*_g_ of 4.12 ± 0.13 nm, and tau187-P301S presents the highest *R*_g_ of 4.55 ± 0.07 nm. Other mutants exhibit intermediate *R*_g_. These data show that a single-point mutation can significantly change the *R*_g_ of the protein, reflecting a change in the conformation ensemble of the different mutants.

In order to confirm that single-point mutations can trigger global conformation rearrangement, we performed native electrospray ion mobility mass spectrometry on tau187-WT and tau187-P301S, which showed the highest difference in *R*_g_ from SAXS experiments (Figure 3A). Proteins with intrinsically disordered regions typically show broad and multimodal charge state distributions, with the relative intensity of higher charge states reflecting the proportion of extended conformations.^18^Figure S4 shows that the charge state distribution of tau187-P301S is slightly shifted toward the higher charge states. We confirmed by ion mobility spectrometry that, in addition, the collision cross section distribution of those higher charge states is shifted toward higher values. This primarily indicates more extended gas-phase conformations, but it has been shown that these reflect solution compactness (collision cross sections are correlated to *R*_g_),^19^ and thus indicate the presence of more extended conformations in the P301S mutants.

We further investigated whether the aggregation propensity could be linked to the structural features of the different mutants. We plotted the aggregation halftime as a function of the radius of gyration *R*_g_ (Figure 3B). Strikingly, we found an excellent correlation (*R*^2^ = 0.93) for the two parameters, demonstrating that the more extended a mutant is, the more aggregation prone it is. The same trend was found when aggregation was triggered with other homonucleotides polyA or polyU (Figure S5E,F). Because the *R*_g_ vs aggregation halftime plots are clustered, we performed additional statistical analysis and determined the minimum number of clusters that can be obtained from the data, using the elbow method (Figure S6). It results that at least 3 to 4 clusters are necessary to properly explain the data, confirming the validity of the regression used in Figure 3B.

## 2.3 Interactions between Tau Molecules Are Repulsive and Modulated by Single-Point Mutations

**Figure 4:**
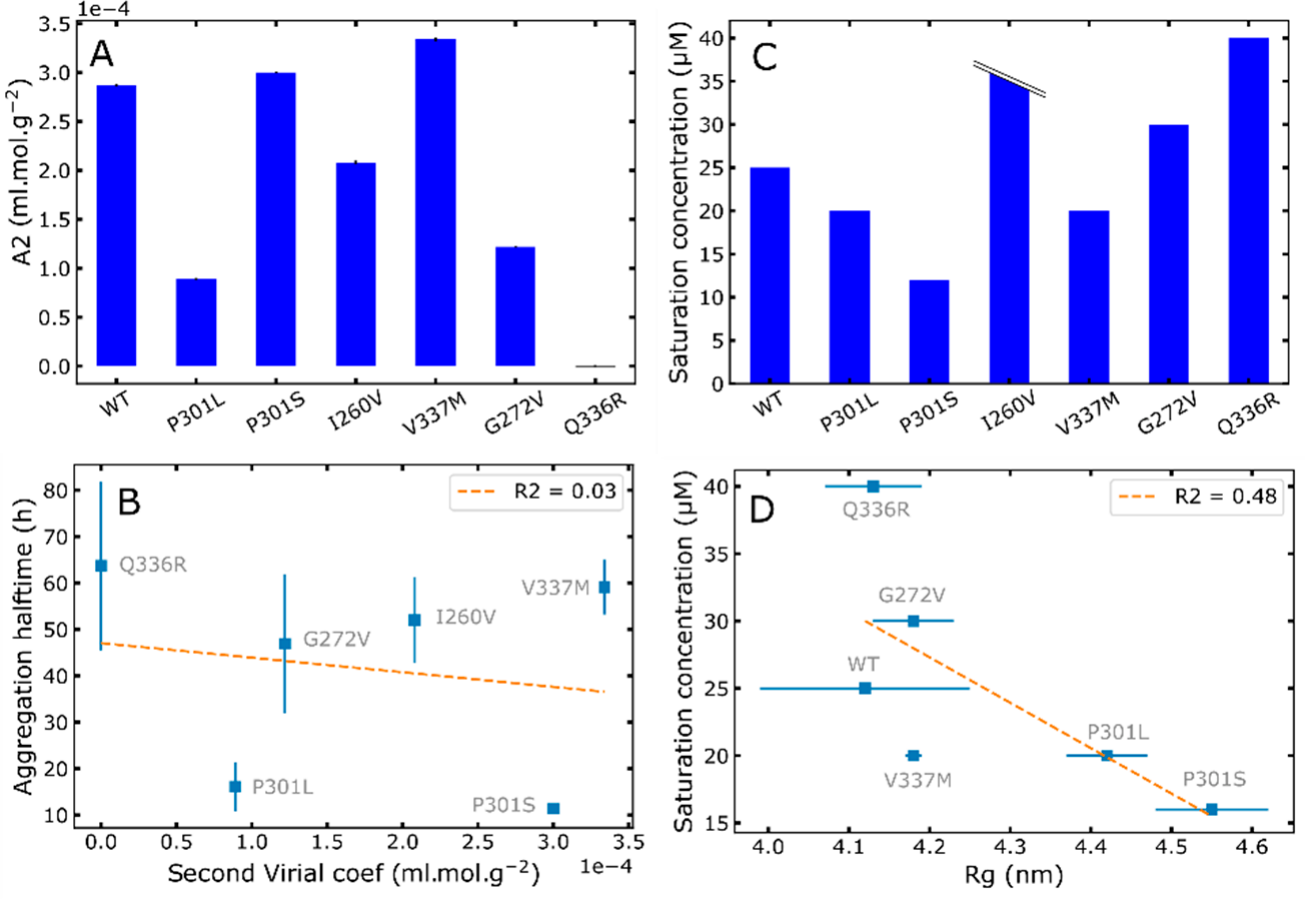
Interprotein interactions modulated by mutations. The second virial coefficient *A*_2_ shows overall repulsive interactions (A). Error bars are from the fitting procedure. Aggregation halftime is not linked to *A*_2_ (B). LLPS saturation concentration *C*_sat_ for different mutants (I260V exceeded the tested maximum concentration of 40 uM) (C). *C*_sat_ is defined as the lowest concentration giving a significant absorption over 3 independent replicates (p-value <0.05; see the “Materials and Methods” section). LLPS saturation concentration correlates with *R*_g_ (D). For correlation plots (B) and (D), WT and I260V data points are not shown because their aggregation halftime and saturation concentration, respectively, are not defined.

Aggregation involves the assembly of many molecules together. Thus, we investigated the nature of interactions between tau molecules in solution. Light scattering from a particle solution intrinsically contains information about the interactions between the particles. Here, we extracted from SAXS experiments the second virial coefficient, *A*_2_, a parameter that reflects interparticle interactions.^20^ The scattering intensities extrapolated at scattering angle *q* = 0 were plotted against protein concentration (Figure S7) and fitted with eq 1 (see “Materials and Methods” section). Positive values of *A*_2_ originate from repulsive interactions (the higher *A*_2_ is, the more repulsion exists), and negative values of *A*_2_ originate from attractive interactions (the more negative *A*_2_ is, the more attraction exists). Figure 4A presents the *A*_2_ for each mutant. *A*_2_ is significantly different for all mutants, revealing that a single-point mutation is sufficient to significantly modulate intermolecular protein interactions. In addition, *A*_2_ is positive for all mutants, except Q336R where it is close to 0, pointing to the fact that interactions are overall repulsive between tau molecules. This is in good agreement with the observation that recombinant tau is extremely stable in solution and did not aggregate in the absence of RNA (Figure S1). Moreover, we evaluated whether the interaction between tau monomers is linked to aggregation propensity triggered by RNA. We found no correlation between *A*_2_ and aggregation halftime (Figure 4B), confirming that aggregation induced by RNA is not directly related to intermolecular protein interactions in solution.

Next, we evaluated the capacity to participate in liquid–liquid phase separation (LLPS), as another parameter reflecting intermolecule interactions. LLPS is a physical process where protein molecules can spontaneously form a high concentration phase relying on a network of interaction between the different molecules.^21^ Tau was previously shown to form LLPS under high salt conditions, where hydrophobic interactions are enhanced.^22^ In a buffer containing 3 M NaCl, we measured the saturation concentration (*C*_sat_), which is the minimum protein concentration at which the formation of LLPS is observed (Figure S8). Figure 4C shows *C*_sat_ values for all mutants. All mutants but I260V exhibited LLPS in the explored range of 0–40 μM. We found that *C*_sat_ is modulated by single-point mutations. tau187-P301L, tau187-P301S, and tau187-V337M are more prone than tau187-WT to form LLPS, suggesting that these mutations favor hydrophobic intermolecular protein interactions. This observation is consistent with MS data where the preferred formation of slightly higher charge states might result from a higher hydrophobicity in the P301S mutant. Indeed, according to the known electrospray ionization mechanisms, higher charge states require some degree of chain ejection from the parent droplets,^23,24^ which can only occur if the protein can sit on the droplet surface, at the water/air interface, and thus if the protein exposes hydrophobic residues.^25^ In contrast, tau187-I260V (which did not form LLPS up to 40 μM), tau187-G272V and tau187-Q336R are less prone than tau187-WT to form LLPS. Furthermore, we found a good correlation between the propensity to form LLPS, as shown by the saturation concentration, and *R*_g_ (Figure 4D). This correlation indicates that more extended conformations facilitate hydrophobic interactions, which are responsible for high-salt LLPS.

## 2.4 Extended Conformations Originate from Enhanced Protein–Water Interactions

**Figure 5:**
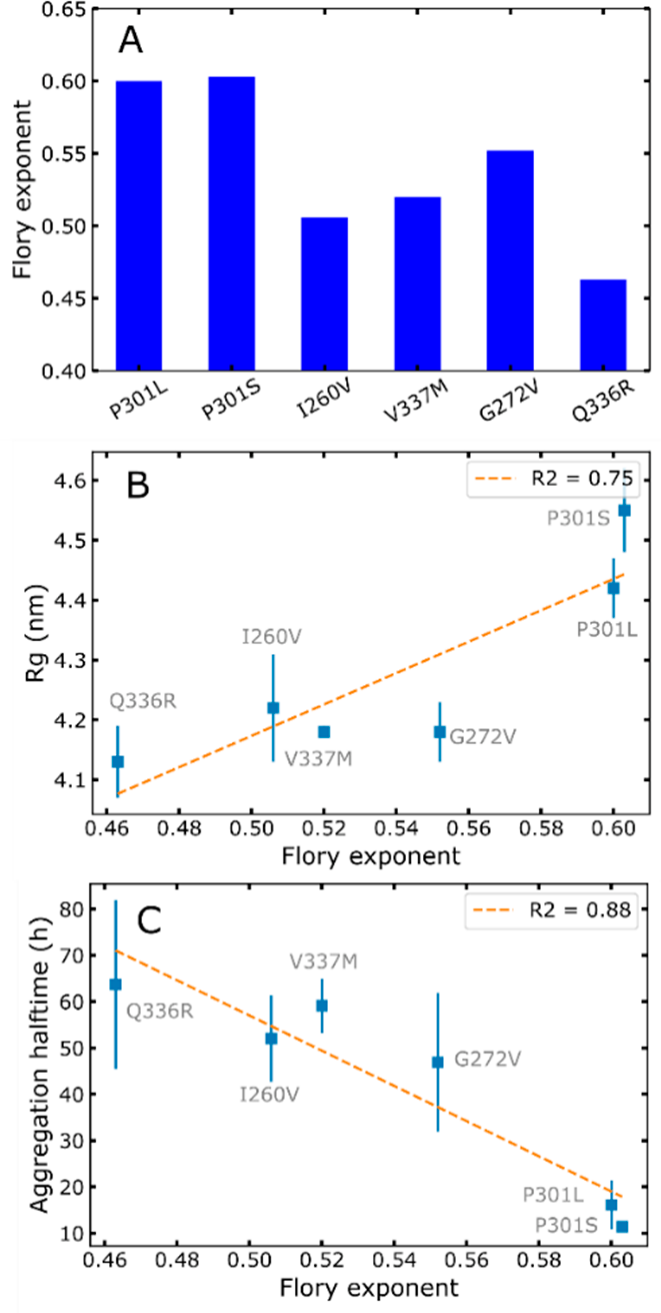
Flory exponent ν of tau187 mutants reveals that single-point mutations modulate tau-solvent interactions (A). *R*_g_ is positively correlated with ν (B), and aggregation halftime is negatively correlated with ν (C).

Then we further investigated the origins of the variation of tau conformations in the different mutants. To do so, we evaluated the contributions of intramolecular protein–protein interactions and protein–solvent interactions, we treated the SAXS data with a previously developed approach aiming at assessing the hydration quality of disordered proteins.^26,27^ From polymer theory, the radius of gyration typically follows the relation *R*_g_ ∼ *N*^ν^, where *N* is the length of the polymer and ν is the Flory exponent. Riback et al. used simulations on model polymers to obtain the relation between *R*_g_ and ν for various solvent conditions.^26^ The polymer molecular form factor and ν were extracted from the SAXS data using the web server made available by Riback et al. (Figure S9). The ν parameter reflects the protein solvation quality for disordered polymers: in the case of an ideal polymer, ν is greater or smaller than 0.5 (theta-solvent condition) for favored or disfavored protein–solvent interactions, respectively. The self-avoiding polymer model would typically present a value of ν = 0.59 for theta-solvent conditions. Recent works have unveiled the presence of localized secondary structure in disordered proteins that tends to increase the Flory exponent.^28,29^ Conversely, disordered proteins also have long-range interactions that tends to make the structure more compact and lower the Flory exponent.^30,31^ Hence, the notion of good and poor solvent should be taken with care with proteins, and a comparison of relative differences between similar sequences in the same buffer is more robust than an interpretation of absolute values. The use of the simulations by Riback et al.^26^ allows to avoid the pitfalls of making too many assumptions on a polymer model and protein–solvent interaction strength. Figure 5A shows ν values for the different mutants. Note that tau187-WT data produced a poor-quality fit, and therefore, its Flory exponent was considered not reliable and was not analyzed (see Figure S10). We observe in Figure 5A that the quality of hydration is significantly modulated by the single-point mutations. P301L/S show the highest ν, which suggests from polymer theory that these mutations favor protein–solvent interactions over protein–protein interactions. The value of ν is furthermore positively correlated to *R*_g_ (Figure 5B). This correlation shows that weaker intramolecular protein interactions (i.e., higher Flory exponent) promote extended conformations and in turn leads to increased aggregation propensity (Figure 5C).

## 3 Discussion

We analyzed the aggregation propensity and structural features of different disease-associated mutants of the tau187 protein fragment. We showed that single-point mutations significantly change the aggregation propensity, as observed by different aggregation halftimes in the presence of RNA as an inducer. We further found that single-point mutations change tau conformations as well as the interactions between monomers. Strikingly we found that the measured *R*_g_ correlates with the aggregation halftime.

**Figure 6:**
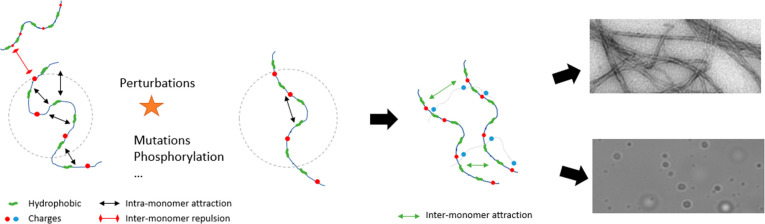
Aggregation model was based on the presented data. In solution, the tau proteins exhibit intermolecular repulsions and does not readily aggregate. Upon modifications, such as mutations, protective intramolecular interactions are released, leading to an increased radius of gyration. These extended conformations are more prone to intermolecular hydrophobic interactions, which in turn drive the formation of amyloid aggregates and LLPS.

FTD-related mutations exhibit little similarities in terms of chemical modifications; some mutations add charges (Q336R) some other remove charges (ΔK280), some change hydrophobicity (P301L) while some are chemically similar (I260V). Thus, the mechanisms of action must rely on subtle disruptions of local or global intramolecular interactions. Early studies suggested that structural effects of disease-associated mutations on tau monomers were small or nonexistent.^6,7,14^ NMR showed subtle local structural changes that were hard to interpret in terms of conformational changes.^14^ A study of the P301L mutation on tau fragments by Chen et al. provided a more comprehensive view of the structural effect of the mutation based on cross-linking mass spectrometry and simulations. They showed that the P301L mutation induces a local opening of the protein, thereby exposing a specific amyloidogenic region of tau. In agreement with this report, here we establish a more general and quantitative link between the differential aggregation propensities of tau mutants and their soluble conformation as probed by its *R*_g_ (Figure 6). This finding is in good agreement with the model where extended conformers are overall favorable for intermolecular contacts of the amyloidogenic region such as PHF6 and PHF6*.

The analysis of the full SAXS curve using a molecular form factor for disordered polymer^26^ (Figures 5 and S6) allowed us to extract the Flory exponent, reflecting the quality of the protein solvation. We show that mutations modulate this parameter. Specifically, some mutants (most drastically P301L/S) exhibit enhanced protein–solvent interactions over intramolecular protein interactions. Although one might intuitively think that favoring solvation quality would stabilize the protein, here we highlight a different mechanism (Figure 6). Rather, P301L/S mutations disrupt protective intramolecular interactions observed before,^15^ thereby leading to increased radius of gyrations and higher aggregation propensities. This mechanism is furthermore consistent with the observation that an increased *R*_g_ correlates with high-salt LLPS propensity (Figure 4D) which relies on hydrophobic interprotein contacts.^22^ Notably, a similar mechanism has been highlighted for α-synuclein,^30^ another amyloid-forming IDP. More generally, this work demonstrates that tau aggregation propensity is encoded, at least partially, in the monomer structure, in agreement with reports on other IDPs such as α-synuclein^30^ and Huntingtin.^32^

Analysis of *A*_2_ showed that overall the tau monomers exhibit repulsive interactions (Figure 4A), which was not expected for an amyloid-forming protein. This observation might explain why recombinant tau hardly aggregates *in vitro* and often requires the use of inducers such as heparin or RNA. The observation that the aggregation halftime induced by RNA does not correlate with A2 shows that RNA completely rewrites this repulsion, likely by compensating for the numerous charges present in the tau protein. One can speculate that cofactor-free aggregation seen for smaller fragments^15,33^ would be more related to this A2 parameter.

Aggregation cofactors induce specific pathways of fibrillization for tau.^34^ RNA acts as a “mild” cofactor^35^ so that the protein still needs to overcome a significant energy barrier to form amyloid, leading to a significant lag time (Figure 2A). Herein we show that increasing the population of aggregation-prone conformers, characterized by lower intramolecular affinity and increased *R*_g_, reduces this lag time and, therefore, the energy barrier to form ThT active species. The impact of mutations is not expected to be similar for other conditions or cofactors, such as heparin, that completely suppress this lag time. Indeed, we verified that there is no significant lag time for the different mutants incubated with heparin and that aggregation halftime does not correlate with *R*_g_ (Figure S11). In this fast kinetics without the lag phase, the aggregation halftime is dominated by the growth rate, which we conjecture to be very dependent on the detailed properties of the tau–cofactor interactions. This view is in line with a recent report showing that the effect of mutations on aggregation is inducer dependent.^36^

A direct correlation between the aggregation halftime and *R*_g_ lays the ground for a rapid and convenient way to evaluate tau aggregation propensity. From SAXS measurements, one can obtain *R*_g_, *A*_2_, and ν of different tau variants (e.g., carrying different post-translation modifications or mutations) to obtain an idea of the aggregation propensity of the variant. This is particularly useful as many different combinations of modifications such as phosphorylation are possible and have been shown to have nontrivial effect on aggregation propensity.^37^

## 4 Materials and Methods

### 4.1 Protein Expression and Purification

Tau187, a truncated version of 2N4R (residues 255–441) was engineered with a poly histidine tag at the N-terminal end. Mutants of tau187 were prepared using site-directed mutagenesis.

The expression and purification of tau187 variants have been previously reported.^38,39^ Genes were transformed into *E. coli* BL21(DE3) that grew at 37 °C, 200 rpm, with addition of 10 μg/mL kanamycin, until reaching optical density (600 nm) of 0.6. Expression was induced by incubation with 1 mM isopropyl-ß-d-thiogalactoside for 2–3 h. Cells were harvested with centrifugation at 5000 *g* for 20 min. Cell pellets were resuspended in lysis buffer (Tris-HCl, pH = 7.4, 100 mM NaCl, 0.5 mM DTT, 0.1 mM EDTA) with 1 Pierce protease inhibitor tablet (Thermo Scientific, A32965), 1 mM PMSF, 2 mg/mL lysozyme, 20 μg/mL DNase, and 10 mM MgCl_2_ (10 mM), and incubated on ice for 30 min. Samples were then frozen and thawed 3 times using liquid nitrogen, then centrifuged at 10,000 rpm for 10 min. 1 mM PMSF was added again, and samples were heated at 65 °C for 12 min and cooled on ice for 20 min. Cooled samples were then centrifuged at 10,000 rpm for 10 min to remove the precipitant. The resulting supernatant was loaded onto a column prepacked with 5 mL of Ni-NTA resins (cytivia HisTrap HP) using an Akta pure system. The column was washed with 25 mL of buffer A (20 mM sodium phosphate, pH = 7.0, 500 mM NaCl, 10 mM imidazole, 100 μM EDTA), 25 mL of buffer B (20 mM sodium phosphate, pH = 7.0, 1 M NaCl, 20 mM imidazole, 0.5 mM DTT, 100 μM EDTA), and 25 mL of buffer A. Protein was eluted with 0–100% gradient over 50 mL of buffer C (20 mM sodium phosphate, pH = 7.0, 0.5 mM DTT, 100 mM NaCl, 300 mM imidazole). Eluents were analyzed by SDS-PAGE to collect the pure fractions. Proteins were then buffer exchanged into working buffer of 20 mM HEPES, 100 mM NaCl, pH 7.0.

### 4.2 SAXS Experiments

The SAXS experiments were conducted on the BM29 beamline at the European synchrotron radiation facility (ESRF).^40,41^ Following two different procedures.

The first experiment was performed in batch, by loading 50 μL of sample in the dedicated BM29 sample changer after centrifugation at 10,000 rpm for 10 min. For each sample measured, a series of 20 frames were acquired with an integration time of 1 s for each frame. The azimuthal integration of the images was done automatically from the beamline control software, and the 1D scattering curves were subsequently used for data analysis. These data sets were used to extract the radius of gyration and the Flory exponent.

In a second experiment, a size-exclusion chromatography (SEC) S200 column of 3 mL volume was used to separate possible contaminants and aggregates from the monomers. The buffer used was 20 mM ammonium acetate, 100 mM NaCl and 5 mM TCEP. The samples were spin downed at 10,000 rpm for 10 min before the injection on the column. The flow rate was set to 0.3 mL/min, and the injected volume was 50 μL. The images were acquired on a Pilatus 2 M detector at a distance of 2.869 m from the sample. Each image is obtained from a 0.5 s exposure of the sample to an X-ray beam. These data were used to extract the A2 parameter using the online UV–vis spectrophotometer to extract protein concentration and directly obtain *I*(*c*, 0).

### 4.3 SAXS Data Treatment and Analysis

The radius of gyration (*R*_g_) was obtained from the linearized batch SAXS data, ln(*I*(*q*)) vs *q*^2^, fitted using the following relation . To obtain a reliable value of *R*_g_, the fit is performed on multiple subdivisions of the fitting region. The subdivision size ranges from 6 points to the full *q*-region used for the fit. The obtained *q*-values are then plotted on a frequency histogram, and the final *R*_g_ is obtained by taking the weighted-average of the histogram. The quality of the *R*_g_ can be assessed by inspection of the histogram, where a proper linear Guinier region should give a narrow distribution of *R*_g_ around the mean value. The obtained *R*_g_ values were compared with values obtained from other software (ATSAS and Riback and Sosnick’s web server)^26,27^ and showed good agreement.

The Flory exponent was obtained by fitting the frames using an χ^2^-type distance from the average of all frames. All the frames that deviated from 2σ, σ being the standard deviation of χ^2^ distances, were eliminated. The buffer subtraction was then performed and the subtracted data were used as input for the Riback and Sosnick’s web server.^26,27^

The SEC-SAXS data contain UV measurement as a function of time along with the X-ray scattering images. The frames that pertain to the UV peak were manually selected as well as the frames that contain only the buffer. The buffer frames are chosen such that they are positioned in time just before the sample frames, which gives the best buffer subtraction (Figure S12). The SEC-SAXS data were used to compute the second virial coefficient, *A*_2_, according to the following:
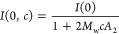
1where *I*(0, *c*) is the extrapolated SAXS intensity at *q* = 0 Å^–1^ (obtained from a Guinier fit at low *q*) and at protein concentration *c*, and *M*_w_ = 20 570 Da is the protein molecular weight. The concentration was obtained from the UV signal recorded after the SEC by dividing the absorbance at 280 nm by the protein molar extinction coefficient ε = 2,800 M^–1^ cm^–1^. The eq 1 was fitted to the data using the Python Scipy’s *curve_fit* routine.

Scripts for SAXS data analysis are deposited and are available on Github (DOI 10.5281/zenodo.7893438).

### 4.4 LLPS Experiments

Different concentrations (0–40 μM) of tau187 mutants were incubated with 3 M NaCl in a 384-well low-volume microplate. The total volume was 30 μL in each well. Each condition was prepared independently in three different wells. Standard deviations over the three wells are presented as error bars. Absorbance at 500 nm was measured in a BMG fluoroStar Omega after 10 min of incubation. LLPS was not detected in the range 0–12 μM protein so the absorbances measured at 0, 4, 8, and 12 μM protein were used to define an absorption baseline. A *t* test was performed between the absorption of these 4 concentrations and each of the upper concentration. The lowest protein concentration giving *p* < 0.05 was reported as the saturation concentration. Raw data are shown in Figure S8 for each mutant.

### 4.5 ThT Experiments and Data Fitting

The tau protein was incubated at 20 μM in 384-well low-volume microplate with 20 μM ThT. The RNA polyC (Sigma P4903), polyA (Roche 10108626001) or polyU(Sigma P9528) were added at 200 μM. Heparin (Sigma H6279) was added at a concentration of 5 μM in supplementary experiments (Figure S11). The working volume was 20 μL. The fluorescence was bottom read in a BMG fluoroStar Omega instrument with excitation and emission wavelengths of 440 and 480 nm, respectively. Each condition was prepared independently in three or two different wells.

ThT kinetic curves were first normalized between 0 and 1. They were then fitted using the *curve_fit* python function with the following equation:
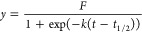
2where *F* represents the final fluorescent intensity, *k* represents the growth rate, and *t*_1/2_ represents the aggregation halftime. Each replicate was fitted independently. The presented error bars on the aggregation halftimes represent the standard deviation over the output *t*_1/2_ obtained from the fit of each replicate. The fitting functions are shown in Figure S3. tau187-WT signal was normalized with the maximum intensity of tau187-P301L for visualization in Figure 2A, since it did not increase.

### 4.6 Electron Microscopy

The carbon film 300 mesh copper grids are hydrophilized by UV light for 10 min. Four μL of samples were applied on the grid for 2 min before applying the staining solution. 4 μL of 2% Uranyl Acetate filtered were applied on grid twice for 1 min. Samples were imaged by a Philips CM 120 transmission electron microscope.

### 4.7 Native Electrospray Ion Mobility Mass Spectrometry

The proteins were buffer exchanged against 100 mM ammonium acetate (prepared with water from Biosolve (UPLC-MS grade)) using a Zeba column (Thermo Scientific). The protein was then diluted to 7 μM in 100 mM NH_4_OAc for the mass spectrometry analysis. Experiments were performed on an Agilent 6560 DTIMS-Q-TOF instrument (Agilent Technologies, Santa Clara, CA), with the dual-ESI source operated in positive ion mode. A syringe pump flow rate of 190 μL/h was used. Capacitance diaphragm gauges are connected to the funnel vacuum chamber and to the drift tube. An in-house modification to the pumping system allows better equilibration of the pressures: an Edwards E2M40 vacuum pump (Edwards, UK) is connected to the source region with two Edwards SP16K diaphragm valves connected to the front pumping lines, while an Edwards nXR40i vacuum pump is connected to the Q-TOF region. The helium pressure in the drift tube was 3.89 ± 0.01 Torr, and the pressure in the trapping funnel was 3.80 ± 0.01 Torr. The pressure differential between the drift tube and the trapping funnel ensures that only helium is present in the drift tube. The acquisition software version was B.09.00. All spectra were recorded by using soft source conditions. The tuning parameters of the instrument (electrospray source, trapping region and post-IMS region (QTOF region)) are optimized for softness as described elsewhere.^42^ The source temperature was set at 220 °C, and the source fragmentor voltage was set to 320 V. The trapping time was 1000 μs, and release time was 200 μs. Trap entrance grid delta was set to 2 V.

Step-field experiments (five drift tube voltages for each sample) were performed to determine the collision cross sections (CCS). The arrival time *t*_A_ is related to ΔV (voltage difference between the entrance and the exit of the drift tube region) by
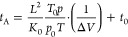
*t*_0_ is the time spent outside the drift tube region and before detection. A graph of *t*_A_ vs 1/Δ*V* provides *K*_0_ from the slope and *t*_0_ as the intercept. The drift tube length is *L* = 78.1 ± 0.2 cm; the temperature is measured accurately by a thermocouple (*T* = 297 ± 1 K), and the pressure is measured by a capacitance gauge (*p* = 3.89 ± 0.01 Torr). The CCS is determined using
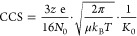
The relative combined standard uncertainty on the CCS of the peak center is ∼2.0%.^43^ The reconstruction of the experimental CCS distributions from the arrival time distributions at the lowest voltage is then performed using equation:^44^
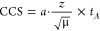
where the factor *a* is determined from the *t*_A_ of the peak center at the lowest voltage and the CCS calculated from the regression described above, from the peak centers.

## Data Availability

Scripts for SAXS data analysis are deposited and available on Github (DOI: 10.5281/zenodo.7893438). SAXS data acquired at the European Synchrotron Radiation Facility (ESRF) are stored under DOI: 10.15151/ESRF-ES-771376723. All other data, data analysis scripts and figure-making scripts are available under DOI: 10.5281/zenodo.7986281.

## Supporting Information Available

Additional data and complementary plots detailing the data treatment procedures (PDF)
