# Correction to “Mutations in Tau Protein Promote Aggregation by Favoring Extended Conformations”

**DOI:** 10.1021/jacsau.5c01194

**Published:** 2025-10-03

**Authors:** Kevin Pounot, Clara Piersson, Andrew K. Goring, Frédéric Rosu, Valérie Gabelica, Martin Weik, Songi Han, Yann Fichou

The following sentence should be added to the Acknowledgments:

We acknowledge the European Synchrotron Radiation Facility for provision of synchrotron radiation facilities and we would like to thank Dr. Stephanie Hutin for assistance in using beamline.

The updated Acknowledgments are as shown below.

