# Supplementary material for: Mutations in Tau Protein Promote Aggregation by Favoring Extended Conformations
Source: JACS Au. 2023 Dec 19;4(1):92–100. doi: 10.1021/jacsau.3c00550 (PMC10806773; doi:10.1021/jacsau.3c00550)
Supplement: Supplementary file 1 — au3c00550_si_001.pdf [file au3c00550_si_001.pdf]

Mutations in tau protein promote aggregation by favoring extended conformations

Kevin Pounot [1], Clara Piersson [2], Andrew K. Goring [3], Frédéric Rosu[4], Valérie Gabelica[4,5], Martin Weik [1], Songi Han [6,7], Yann Fichou [2]*

1: Univ. Grenoble Alpes, CEA, CNRS, Institut de Biologie Structurale, 38000 Grenoble, France

2: Univ. Bordeaux, CNRS, Bordeaux INP, CBMN, UMR 5248, 33600 Pessac, France

3: Department of Chemistry and Biochemistry, University of California Los Angeles, Los Angeles CA 90095, USA

4: Univ. Bordeaux, CNRS, INSERM, IECB, UAR3033, US01, F-33600 Pessac, France

5: Univ. Bordeaux, CNRS, INSERM, ARNA, UMR5320, U1212, IECB, 33600 Pessac, France

6: Department of Chemical Engineering, University of California Santa Barbara, Santa Barbara CA 93106, USA

7: Department of Chemistry and Biochemistry, University of California Santa Barbara, Santa Barbara CA 93106, USA

*y.fichou@iecb.u-bordeaux.fr

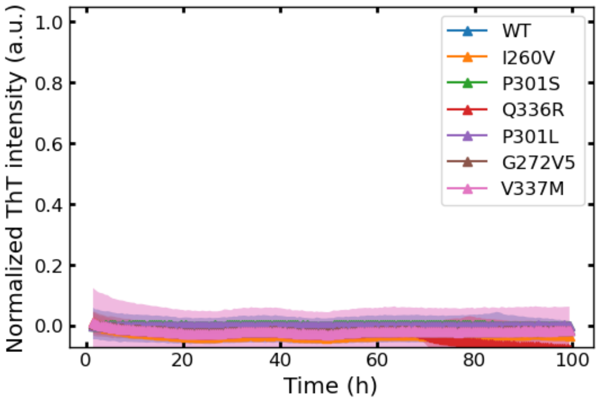

Figure S1 : None of the mutants showed aggregation after 4 days of incubation at 37 °C.

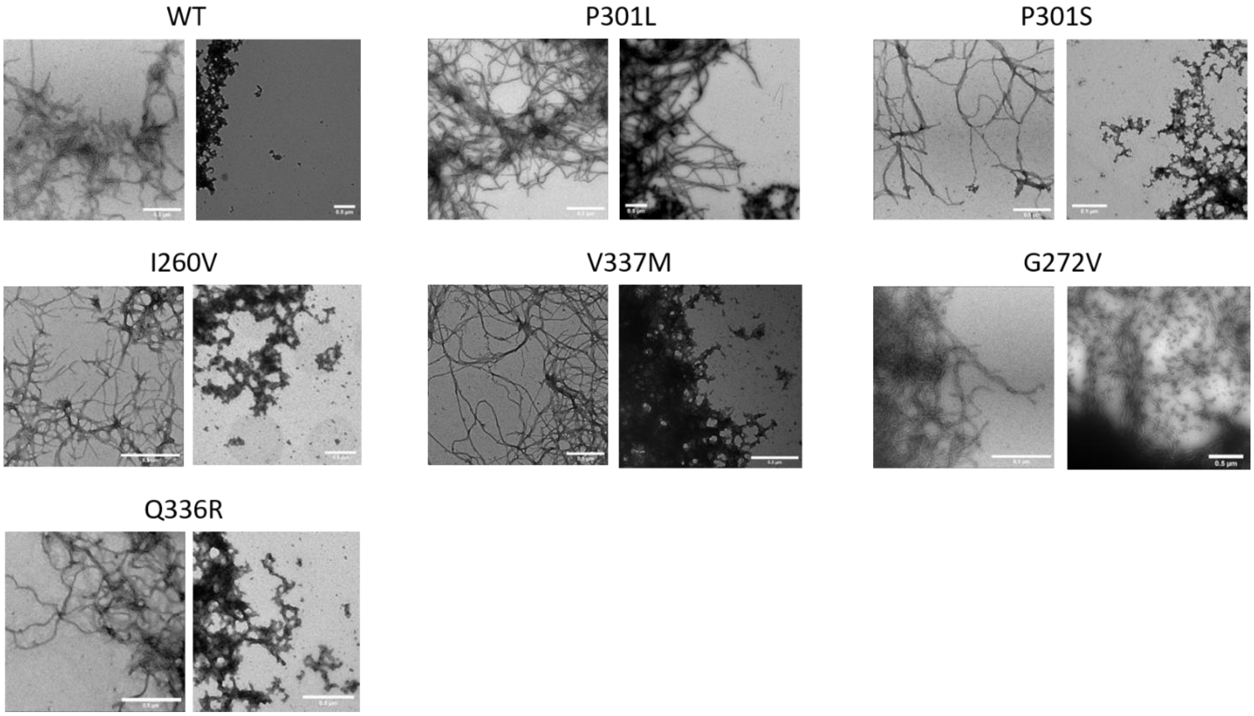

Figure S2 : TEM images for tau187 (20 μM) mutants incubated with heparin (5 μM) for 24h (left) or polyC (200 μg/ml) for 100h (right). In general, tau187 incubated with polyC resulted in a mixture of amorphous and filamentous aggregates. Scale bars are 0.5 μm.

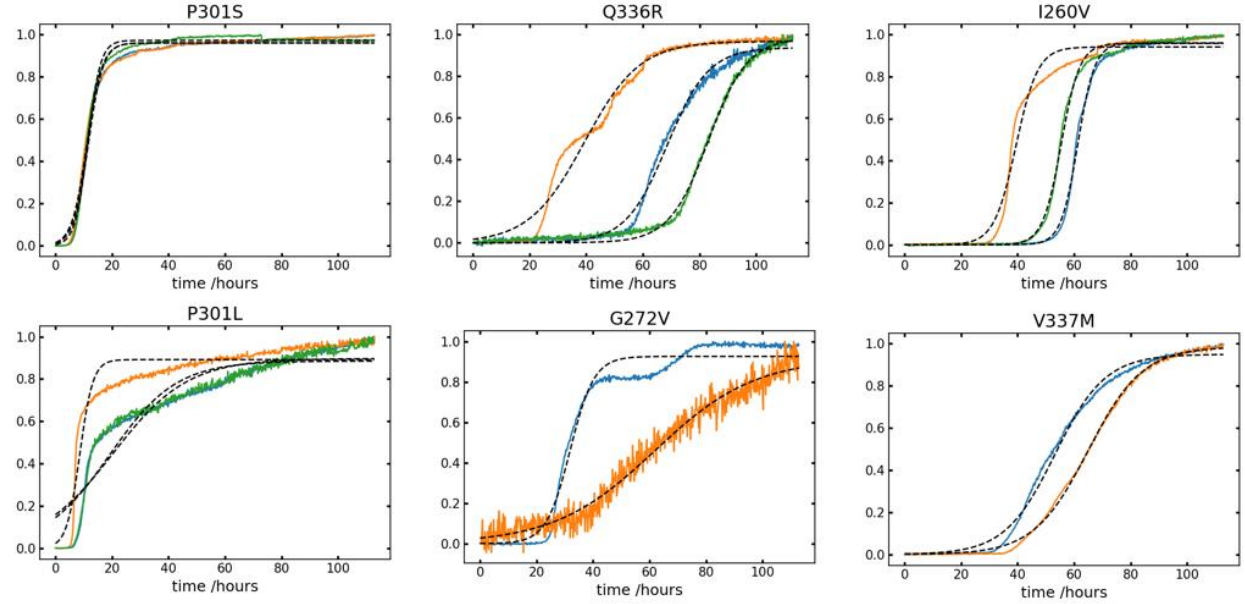

Figure S3: Aggregation kinetics followed by ThT fluorescence for each mutant incubated with RNA polyC. The fit used to derive aggregation halftime from equation (2) in figure 2 are showed as dash lines. Tau187-WT was not fitted as it showed no increase in fluorescent.

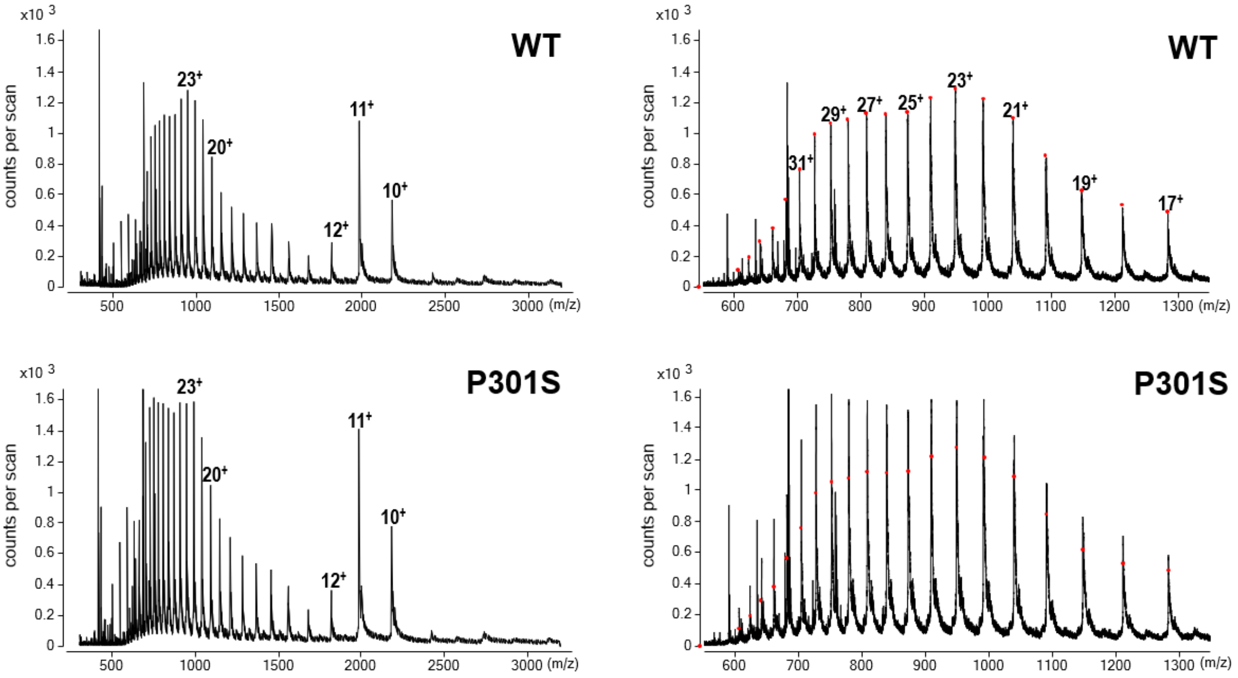

Figure S4 : Native electrospray mass spectra of wild type tau and mutant P301S obtained using soft instrumental conditions. The concentration of the protein was 7 µM in 100 mM ammonium acetate. The spectra on the right-hand side show a zoom on the charge distribution of high charge states, and the dots indicate the distribution of the WT. All charge states are more intense for P301S, and the charge state distribution is more displaced to high charge states (>25+).

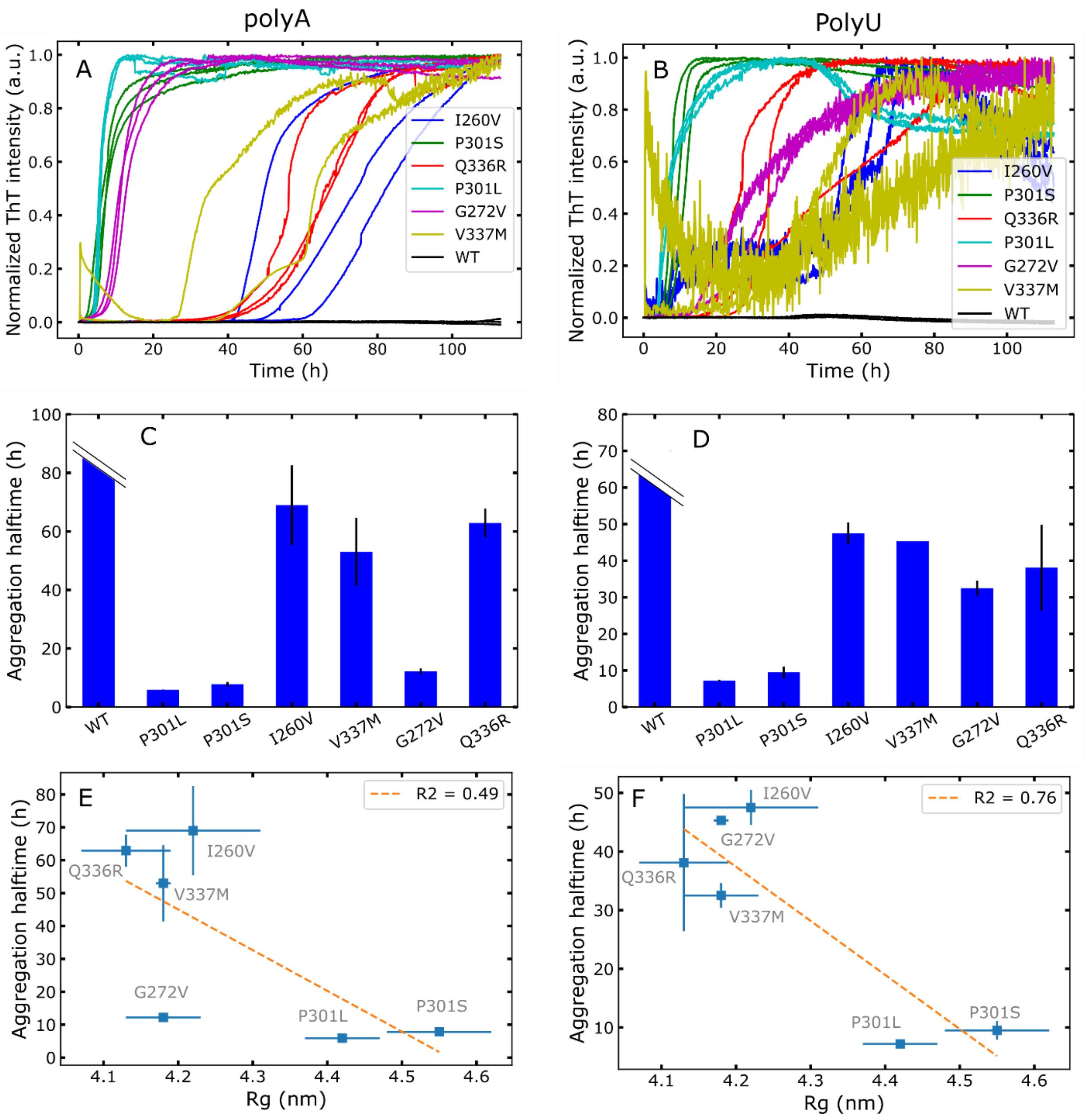

Figure S5: ThT fluorescence as a function of time for different mutants of tau187 incubated with RNA polyA (A) and polyU (B). Protein and RNA concentrations were 20 μM and 200 μM, respectively. The curves are normalized between 0 and 1. Aggregation half time for each mutant incubated with polyA (C) or polyU (D), extracted from a fit of each ThT curve. Error bars represent the standard deviation over the half times obtained from the different replicates. The correlation of aggregation halftime and the radius of gyration is shown in panels (E) and (F).

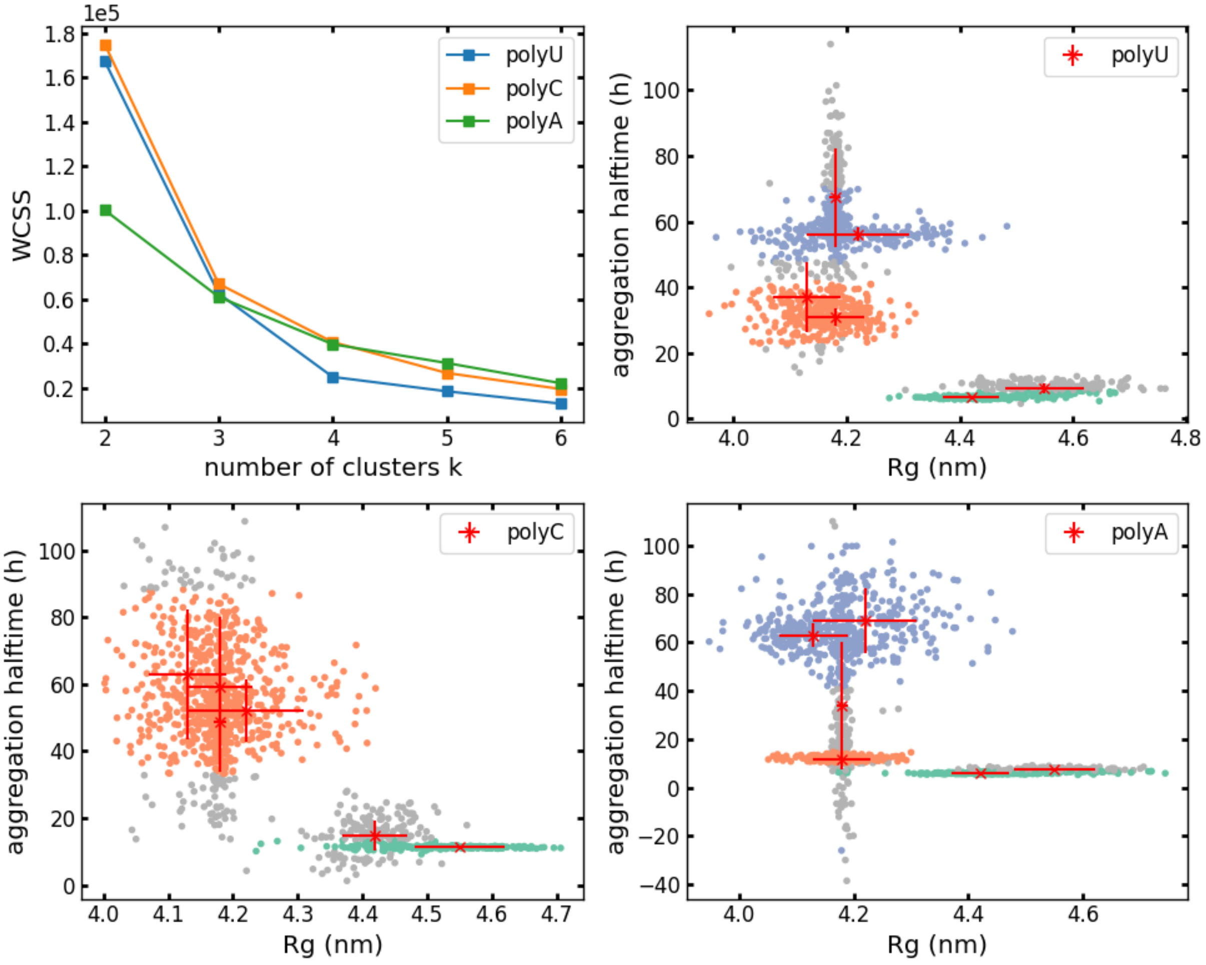

Figure S6: Cluster analysis on experiment-based reconstructed data. The data from Figure 3 were used to generate 2D distributions of 200 points per experimental value following a Gaussian distribution $(X_i, Y_i) \sim N(\mu, \sigma)$, where μ and σ are taken from the experimental average and standard errors, respectively. Using this set of points, KMeans clustering was used using the *Scikit-learn* Python package to determine the minimum number of clusters that should be used to properly explain the data. In that case, the within-cluster sum-of-squares (WCSS) metric was used and plotted against the number of clusters for different RNA **(A, blue - polyU, orange - polyC, green - polyA)**. In addition, the OPTICS algorithm from *Scikit-learn* was used with *min_samples* argument set to 100. The results are plotted for polyU **(B)**, polyC **(C)** and polyA **(D)** as dark blue points for outliers and other colors depending on the cluster they pertain to. The original points with their associated error bars are represented in red.

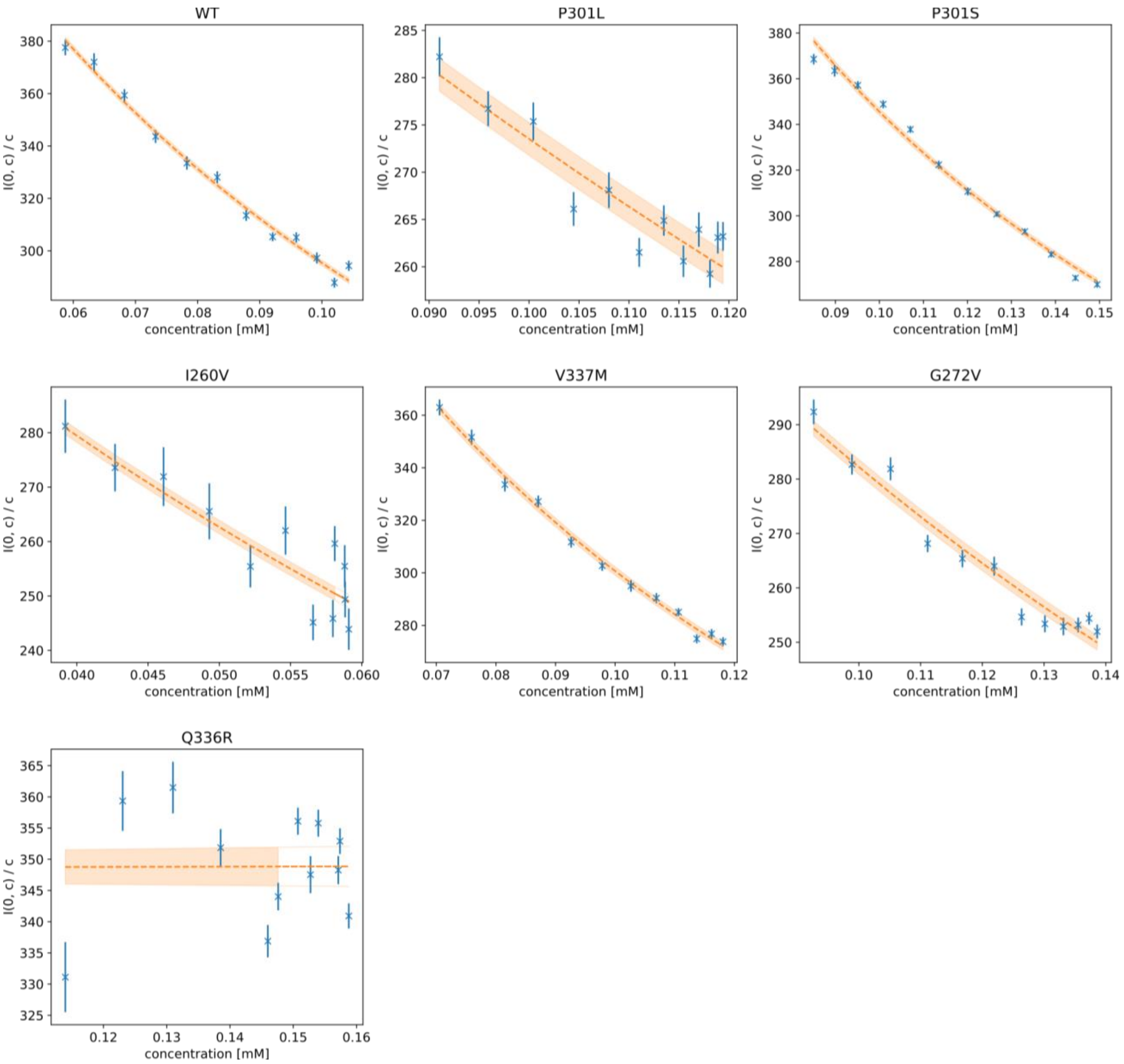

Figure S7: Fits of I(0) Vs concentration to extract A_2. The SEC-SAXS data were treated and analyzed as described in Methods. On each panel, the corresponding mutant is given on top and the extrapolated signal I(0, c) / c (blue crosses with error bar) is plotted as the function of concentration c. The fit of equation 1 is plotted (orange dashed curve) with a confidence interval. The orange area around the fitted curve is the uncertainty on the model computed using:

$$\sigma_f^2 = \left(\frac{\partial f}{\partial I0}\right)^2 \sigma_{I0}^2 + \left(\frac{\partial f}{\partial A_2}\right)^2 \sigma_{A_2}^2 + 2\,\frac{\partial f}{\partial I0}\frac{\partial f}{\partial A_2}\quad Cov(I0, A_2) \tag{2}$$

where f is the fitting function from equation 1, σ_{I0}^2 and $\sigma_{A_2}^2$ are the estimated errors on the fitted parameters for I0 and A_2, respectively and Cov(I0, A_2) is the covariance function.

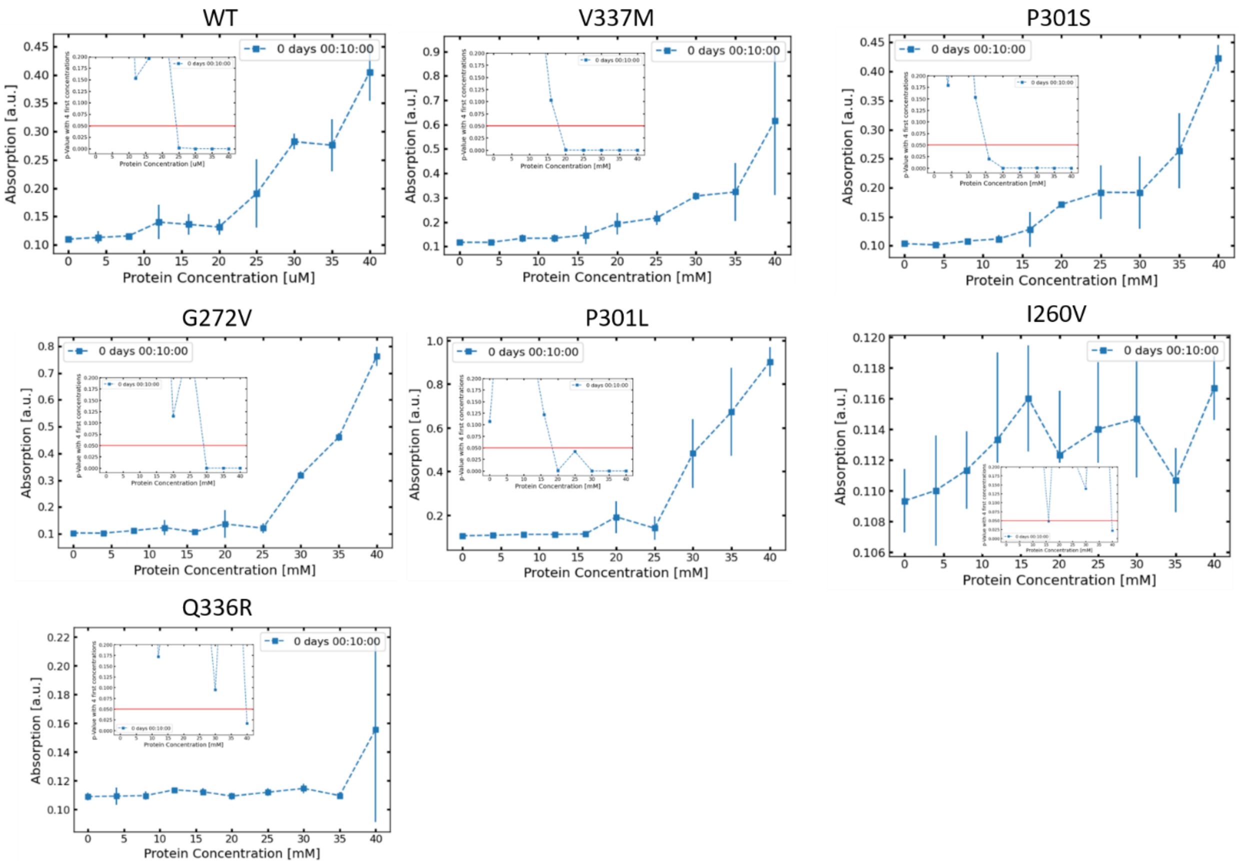

Fig. S8: LLPS are formed at 3M NaCl and probed by absorption. Raw absorption data as a function of protein concentration are shown for all mutant. (inset) T-test used to defined the saturation concentration for LLPS formation.

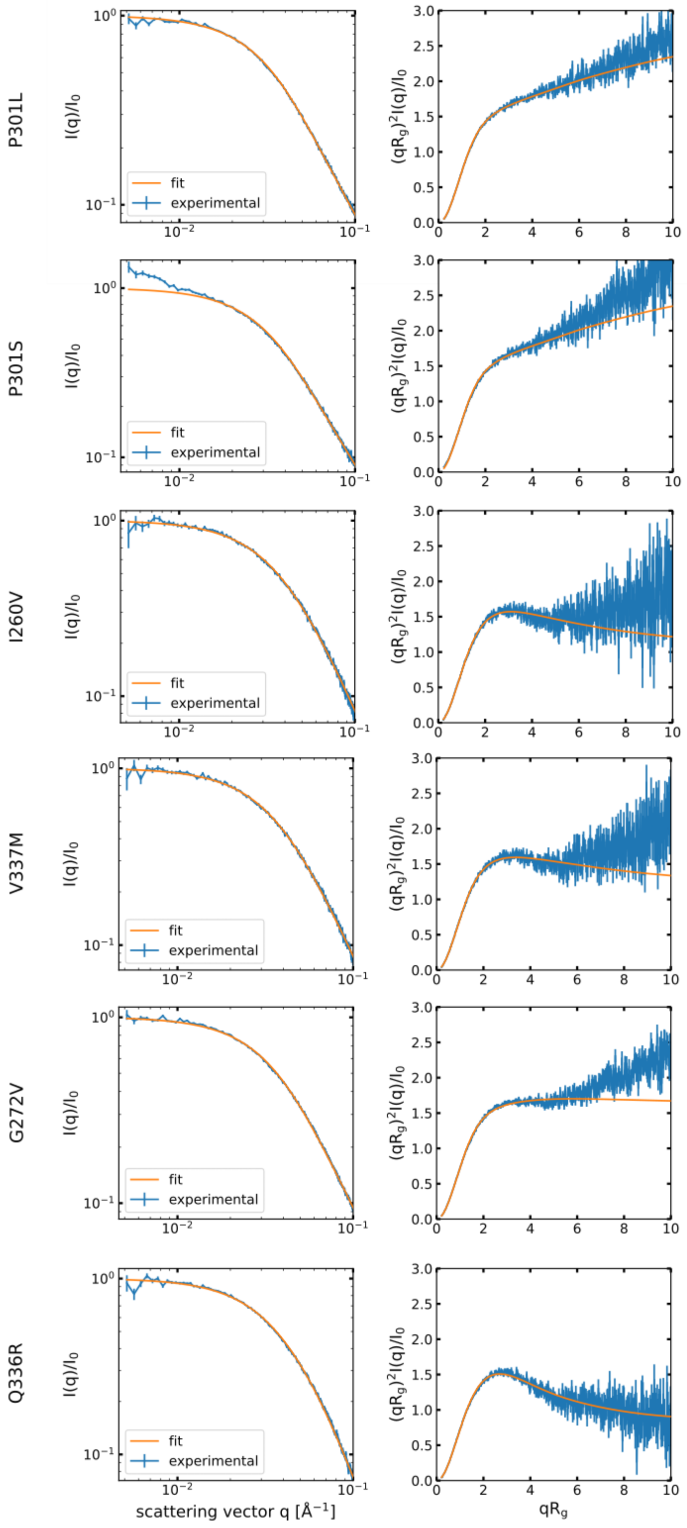

Figure S9: Best fit of the SAXS data obtained from the server http://sosnick.uchicago.edu/SAXSonIDPs.

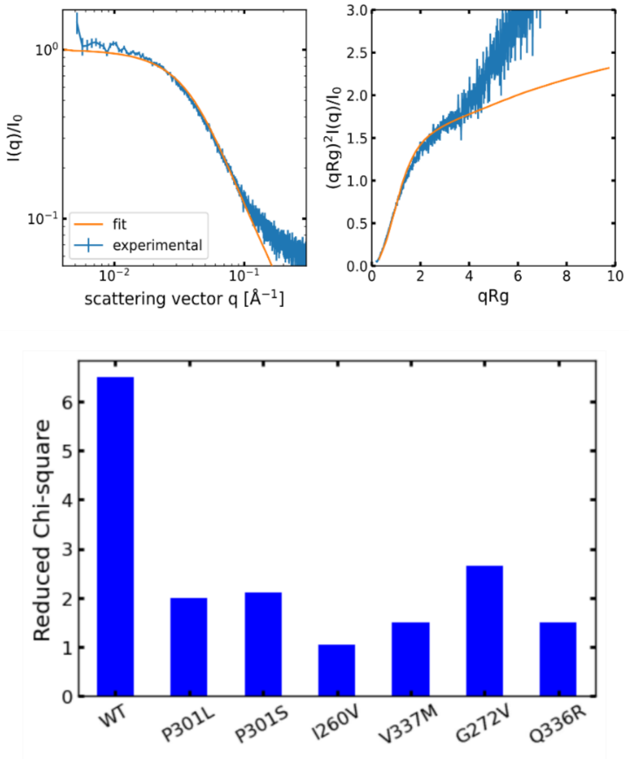

Figure S10: (Upper panel) Best fit of tau187-WT data from the server http://sosnick.uchicago.edu/SAXSonIDPs. (Lower panel) Reduced chi-square for the fit of the SAXS of each tau mutant. Because the fit was poor for tau187-WT, the output parameters (Flory exponent) were not analyzed in manuscript.

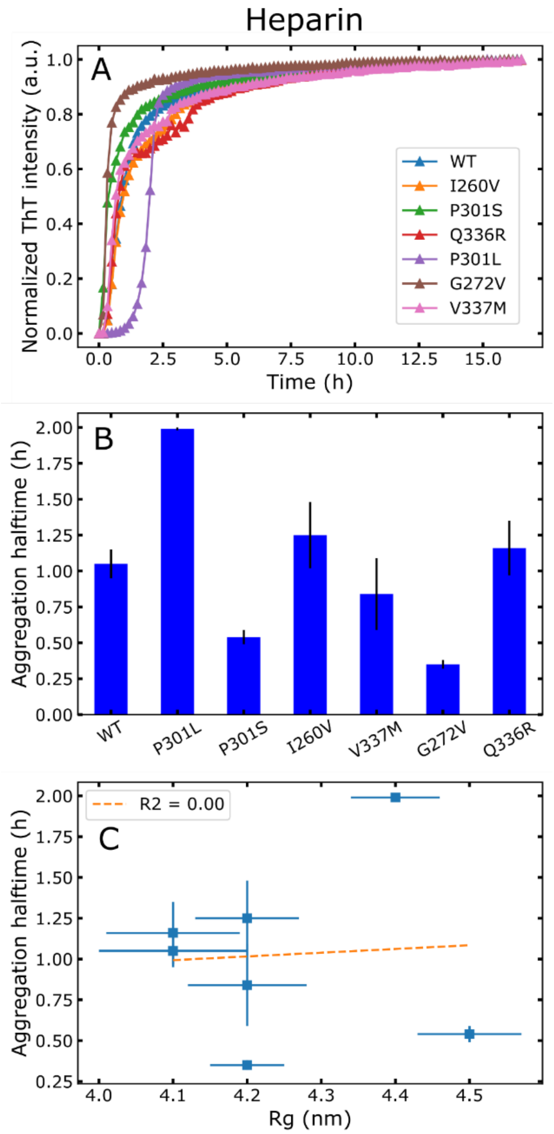

Figure S11 : Heparin suppresses aggregation lag time for almost all mutants, including tau187-WT (A). Aggregation halftime (B) does not correlate with Rg (C).

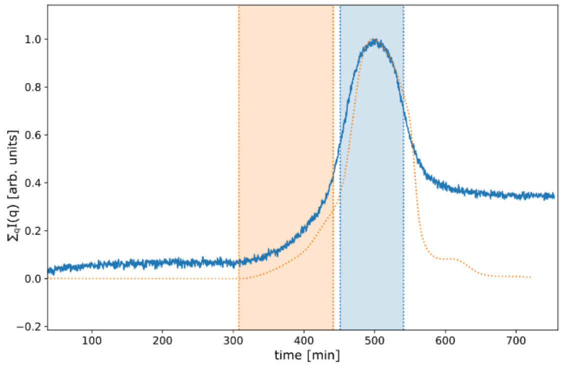

Figure S12: Typical selected regions for the SEC-SAXS data. The total scattering signal is plotted with a solid blue line, the UV measurement is plotted with a dotted orange line with a time delay such that it takes into account the time for the sample to flow from the column output to the X-ray capillary. The selected frames that are averaged for the sample are represented by the blue area and the frames used for the buffer by the orange area.
